# Humidity-driven lactose crystallization on milk powders: a surface-level study

**DOI:** 10.1016/j.crfs.2026.101465

**Published:** 2026-06-05

**Authors:** Jennifer Burgain, Elise Germain, Ummuhani Ugur, Serafim Bakalis, Mogens Andersen, Anni Bygvrå Hougaard, Alireza Naseri, Kirsten Gade Malmos, Claire Gaiani

**Affiliations:** a Université de Lorraine, LIBio, Nancy, F-54000, France; b Department of Food Science, University of Copenhagen, Frederiksberg, 1958, Denmark; c Lactosan A/S, Ringe, 5750, Denmark; d Arla Foods Amba, Aarhus, Denmark; e Institut Universitaire de France (IUF), Paris, France

**Keywords:** Milk powder, Surface, Crystallization, Glass transition temperature, Humidity

## Abstract

This study introduces *in-situ* atomic force microscopy (AFM) under controlled humidity ramps as a novel approach for real-time monitoring dairy powder surface evolution. Applied to skim milk powder (SMP) and whole milk powder (WMP) as case-study matrices, the method was combined with dynamic vapor sorption (DVS), X-ray photoelectron spectroscopy (XPS), differential scanning calorimetry (DSC), and powder rheology to provide a multi-scale characterization of humidity surface changes. Both powders exhibited sigmoidal sorption isotherms with a critical transition (40–60% RH) associated with moisture-induced plasticization of amorphous lactose. *In-situ* AFM revealed limited surface changes below 43% RH, followed by a sharp increase in surface roughness above 53% RH consistent with surface restructuring likely associated with lactose crystallization. This restructuring occurred earlier and more extensively in SMP, with rapid roughness growth, while WMP showed delayed and attenuated surface changes possibly due to its lipid-rich surface and its influence on water accessibility and/or lactose mobility. XPS analyses confirmed surface rearrangements with a C/O ratio decrease compatible with lactose crystallization. Increased RH significantly impaired powder flowability, particularly above 60% RH.

## Highlights

• Lactose crystallization-related surface changes were detected between 40 and 60% RH in milk powders.
• In situ AFM revealed surface roughness increase above 53% RH, earlier in SMP than WMP.
• Lipid-rich surface of WMP may delay water diffusion and/or restrict lactose mobility, attenuating crystallization-related surface restructuring.
• XPS confirmed humidity-driven surface rearrangements with a decreasing C/O surface ratio consistent with lactose crystallization.
• Above 60% RH, powder flowability deteriorates, threatening technological performance.

## Graphical abstract

**Figure:**
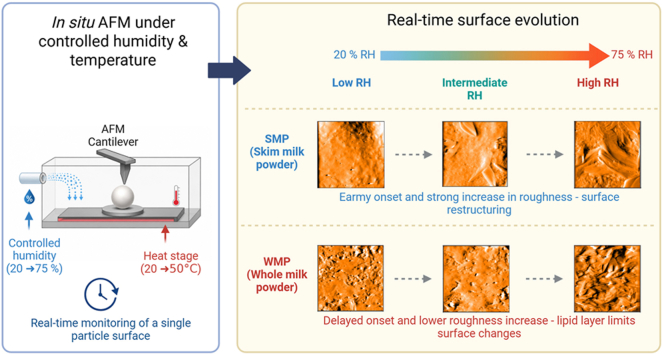

## 1 Introduction

Liquid foods are highly perishable products, and spray-drying has become the method of choice to stabilize liquid ingredients and particularly milk. By removing water from milk, this process prevents undesirable reactions (i.e., lipid oxidation, enzymatic activity, microbial growth) also reduces product volume, thereby facilitating transport. The resulting powders are designed for long-term stability, ensuring extended shelf life with consistent quality (Fang and Bhandari, 2012). Thanks to these advantages, milk powders occupy a central place in the food industry which allows them to be key ingredients in a wide range of formulations. However, despite their stability, they can undergo significant transformations during storage, with consequences for their functional, nutritional and sensory attributes (Ryabova et al., 2023).

Among the phenomena affecting dairy powder quality during storage, humidity-driven changes are significant because they operate across multiple scales simultaneously. It begins with moisture adsorption by the amorphous lactose matrix. As water is absorbed, it acts as a plasticizer, progressively depressing the glass transition temperature (Tg) of the lactose–protein matrix (Roos, 1993). When Tg falls below the storage temperature, the material transitions from a glassy to a rubbery state, increasing molecular mobility. This rubbery, viscoelastic state drives inter-particle bridge formation, particle stickiness, and caking (Fitzpatrick et al., 2007). Subsequent lactose crystallization consolidates these bridges and further alters particle microstructure by generating needle-like surface structures and increasing roughness. Simultaneously, elevated humidity can trigger lipid migration toward the particle surface, promote browning, reduce solubility, and impair reconstitution (Ho et al., 2021; Masum et al., 2020; Gaiani et al., 2026). In whole milk powder (WMP), surface lipids introduce additional complexity as they may modulate water diffusion, may restrict lactose mobility, and may delay both the glass transition and crystallization kinetics (Qí et al., 2025; Saxena et al., 2020; Clark et al., 2016). This complexity of phenomena calls for multi-scale, time-resolved characterization (Phosanam et al., 2020; Burgain et al., 2017).

Because the surface of a particle governs both inter-particle interactions and the initial contact with water during reconstitution, surface characterization is particularly informative. Over the past two decades, dedicated techniques have been developed for the surface analysis of dairy powders (Burgain et al., 2017). Among them, X-ray photoelectron spectroscopy (XPS) quantifies the elemental composition of the outermost layer and has revealed surface enrichment of fat and protein relative to bulk composition, as well as their redistribution under humidity. Scanning electron microscopy has documented morphological changes such as crystal growth, surface cracking, agglomerate formation associated with humidity and/or aging (Phosanam et al., 2020; Maher et al., 2015). Atomic force microscopy (AFM) has proven powerful for capturing nanoscale surface topography and mechanical heterogeneity (Gaiani et al., 2021, 2026). Indeed, AFM studies have reported humidity- and temperature-induced roughness increases, nanomechanical hardening of casein-micelle surfaces, and the emergence of distinct surface domains associated with fat and crystalline structures (Burgain et al., 2017; Gaiani et al., 2021). However, a fundamental limitation shared by essentially all these approaches is due to the fact that samples are characterized after equilibration at a fixed humidity level, not during the process itself. The kinetics of surface reorganization under dynamic humidity conditions remain inaccessible. Bridging this gap requires a real-time, *in-situ* approach capable of tracking individual particle surface evolution continuously as humidity changes.

In this context, the present work addresses this gap by introducing *in-situ* AFM under controlled stepwise humidity ramps. SMP and WMP were selected as contrasting case-study matrices (lactose/protein vs. fat-containing surfaces) to demonstrate the approach across two industrially representative compositions. The principal objective is to establish the feasibility and sensitivity of *in-situ* humidity-controlled AFM for dairy powder surface characterization, and to provide a methodological foundation that future studies can build upon.

## 2 Material and methods

### 2.1 Powders and materials

SMP and WMP were supplied by Arla Foods Ingredients (AFI, Denmark). The chemical composition is presented in Table 1. The fresh powders were received in hermetic packaging and stored at 10 °C. For all experiments unless AFM, the samples were stored in different controlled relative humidity chambers using salt solutions to reach the following RH (23, 43, 53, 58 and 75%). Samples were stored two weeks, at a temperature of 20 ± 2 °C, in the chambers to allow equilibrium and water activity was checked using a WaterLab aw meter (Steroglass, San Martino, Italy).

**Table 1:** Chemical composition of the two industrial powders used in the study (g.100 g^−1^)^a^.

	Fat	Proteins	Lactose	minerals	water
**SMP**	0.5-0.9	32	54	9	3.2
**WMP**	28	24	39	5.9	3.2

a values given by the respective industrial partners (Gaiani et al., 2026).

The consumables used to generate controlled relative humidities were salts with a purity ≥98% (Sigma-Aldrich, Saint Louis, Missouri, USA): potassium acetate, potassium carbonate, magnesium nitrate, sodium bromide and sodium chloride. These salts were used in saturated solutions to reach RH at equilibrium of respectively 23, 43, 53, 58, and 75%. Phosphorus pentoxide was also used as a desiccant to maintain extremely low humidity conditions and prevent moisture adsorption during sample preparation.

### 2.2 Physicochemical properties

#### 2.2.1 Dynamic vapor sorption (DVS)

Sorption isotherms of the powders were acquired with a DVS analyzer (Surface Measurement Systems Ltd., London, UK) equipped with a Cahn microbalance. Measurements were performed at RH levels ranging from 0 to 80%, at a constant temperature of 20 °C. A sample weighing between 10 and 15 mg was placed in the pan and introduced into the chamber. Prior to the experiment, the sample was dehydrated for 300 min at 0% RH. The samples were then subjected to a hydration program with 10% RH increments up to 80%. Equilibrium was considered achieved when the mass change rate (*dm*/*dt*) was less than 0.002 mg min^−1^. Experiments were performed in triplicate for each powder.

#### 2.2.2 Particle size distribution

Particle size distribution was measured using a Mastersizer MS3000 (Malvern Instruments, UK) with the Aero S dry dispersion unit. The measurements were performed under dry conditions. The refractive index of the particles was set to 1.46, while the refractive index of the dispersant (air) was set to 1.00. To ensure appropriate laser obscuration, the analytical conditions were set as follow: air pressure of 2 bars, funnel length of 2 mm, and powder feed rate between 80 and 90%. The classical descriptors were used to estimate the particle size distributions (d10, d50, d90) and to calculate the Span. The span is a polydispersity indicator defined as the width of the distribution normalized by its median value using equation (1).

eq. 1 $$\text{span}=\frac{d90-d10}{d50}$$

Each replicate was carried out using around 10 g sample and obscuration is set between 0.1 and 15% to ensure good analytical accuracy. Two replicates were performed for each powder and under all RH conditions.

#### 2.2.3 Glass transition temperature

Calorimetric measurements on powders were performed using a DSC 250 apparatus from TA Instruments. About 10 ± 2 mg of sample was weighed and sealed in aluminum hermetic pans with a mechanical crimp. The samples were heated under a nitrogen atmosphere from 20 °C to 180 °C at a constant heating rate of 5 °C·min^−1^. Glass transition temperatures (Tg) were extracted at the midpoint from the heat flow-temperature curve. The samples analyzed were SMP and WMP equilibrated at 11, 23, 43, 53, 58, and 75% RH.

### 2.3 Surface characterizations

#### 2.3.1 X-ray photoelectron spectroscopic analysis

Surface chemical atomic percentages (C, O, N, Na, Cl, P) were measured with a KRATOS Axis Ultra X-ray photoelectron spectrometer (Kratos Analytical, Manchester, UK) equipped with a monochromatic Al Kα X-ray (hѵ = 1486.6 eV) operated at 150 W. Samples were placed on the sample holder's surface using adhesive carbon tape and overnight outgassed under high vacuum. All spectra were recorded at 90° take-off angle, the analyzed area being about 700 μm × 300 μm (with approximatively 10 nm depth surface analyze). Survey spectra were recorded with 1.0 eV step and 160 eV analyzer pass energy and the high-resolution regions with 0.05 eV step and 20 eV pass energy (Gaiani et al., 2006). Two measurements were performed for each powder and under all RH conditions.

#### 2.3.2 Atomic force microscopy

The powders were fixed on a round glass (24 mm) using spread thin film epoxy. They were carefully sprinkled on the glass by shaking the spatula. In the case of agglomerated particles, a regulated compressed air was used to eliminate the bulk powders and have clear single particle distribution. Finally, the samples were allowed to dry overnight in a sealed desiccator containing phosphorus pentoxide salt in order to avoid moisture adsorption.

For each humidity ramp, the same individual particle was continuously monitored and characterized by AFM during 420 min. This approach was chosen to directly follow the morphological evolution of a given particle during the humidity ramp. The blue marking horizontal arrows were intentionally retained in the AFM figures to help readers identify the particle and track the successive modifications occurring during the humidity ramp. Five independent particles were each subjected to the complete humidity ramp (20 to 75% RH), yielding five full RH/time profiles from which mean ± SD roughness values were calculated.

• ***Experimental device used to follow in-situ the surface modifications***

**Fig. 1:**
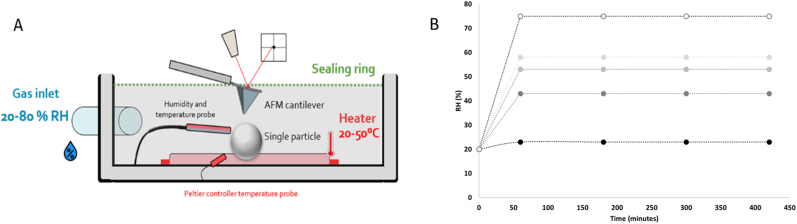
Experimental design in order to follow *in-situ* the particle surface evolution (A) and RH ramps applied from 20 to 23. 43. 53. 58 and 75% RH respectively with a plateau (B) (Badin et al., 2023, 2025).

An environmental AFM system was used to investigate the in-situ evolution of individual particle surfaces under controlled humidity and temperature conditions, following an approach previously applied to maltodextrin (Badin et al., 2023, 2025). The setup consists of a sealed, low-volume chamber equipped with an AFM cantilever for surface characterization, a temperature-controlled heating module (20–50 °C) based on a Peltier system, and a probe placed near the cantilever for continuous monitoring of temperature and relative humidity (Fig. 1A). RH inside the chamber was regulated between 20 and 80% by mixing dry and humidified nitrogen flows (Gianetti et al., 2020). This configuration ensures high measurement stability and maintains the resolution required for topographical analysis. In this study, a single particle of powder (SMP or WMP) was positioned under the AFM cantilever to allow precise *in-situ* characterization of surface changes. Starting from an initial RH of 20% at room temperature (25 ± 2 °C), controlled humidification ramps were applied over 60 min to reach the target humidities (23, 43, 53, 58 or 75%) (Fig. 1B). After 60 min, each relative humidity level was then maintained at a constant plateau for 420 min. AFM topographic images were recorded at different times (60, 180, 300 and 420 min) at each RH level to quantify the progression of the surface. This methodological approach, applied here for the first time to dairy powders, enables precise monitoring of the structural evolution of a single particle exposed to successive humidity conditions.

• ***AFM Imaging***

AFM measurements were performed using a Flex Axiom system and Nanosurf C3000 software (Nanosurf, Liestal, Switzerland). Topography images were obtained by contact mode at 1Hz scan with Stat0.2LAuD cantilevers (resonance frequency 13 kHz) (Nanosurf, Liestal, Switzerland) with a spring constant of 0.2 N/m. The tip used was a pyramidal probe. All roughness calculations were performed using equation (2) to determine the roughness values.

eq. 2 $$Rq=\sqrt{\frac{\sum_{i=1}^{N}{\left(Zi-Zav\right)}^{2}}{N}}$$

Where, Rq, Zav and N are the roughness, average height of the pixels and the number of pixels, respectively.

The standard deviation of the z values determines the root mean square (Rq) roughness.

### 2.4 Functional properties

#### 2.4.1 Powder rheology with the FT4 equipment

Powder flow behavior was characterized using a FT4 powder rheometer (Freeman Technology, Worcestershire, United Kingdom) by means of the Stability and Variable Flow Rate tests. Approximately 10 g of powder was gently introduced into a glass vessel (25 mm internal diameter, 10 mL volume). Prior to testing, the powder bed was conditioned using the FT4 conditioning blade in order to homogenize the sample, minimize the influence of aeration and segregation, and improve measurement reproducibility.

The Stability test was performed to assess the reproducibility and sensitivity of powder flow behavior to repeated mechanical conditioning. The test consisted of multiple consecutive dynamic flow measurements carried out at a constant blade tip speed, with a conditioning cycle applied between each measurement. Conditioned bulk density (CBD, g.mL^−1^), calculated as the ratio of split mass to split volume, was determined to assess powder packing behavior and its propensity to densify under standardized conditioning. The total flow energy recorded during each cycle was used to calculate the Stability Index (SI), defined as the ratio between the flow energy measured during the final cycle and that measured during the initial cycle. An SI value close to unity indicates stable flow behavior with minimal evolution of the powder structure during testing. The Stability test also provided the Basic Flowability Energy (BFE) and the Specific Energy (SE). The BFE corresponds to the total flow energy measured under a defined, moderately stressed flow regime and reflects the resistance of the powder to flow under confined conditions. The SE was determined under low-stress flow conditions and represents the energy required to mobilize the powder during gentle flow, serving as an indicator of powder cohesion.

Following the Stability test, the Variable Flow Rate test was conducted to assess the sensitivity of powder flow behavior to changes in flow rate. Dynamic flow measurements were performed at different blade tip speeds, and the flow-rate-related index (FRI) was calculated as the ratio of flow energies measured at different flow rates. The FRI provides information on the dependence of powder flow behavior on shear rate and processing conditions.

Two measurements were performed for each powder and under all RH conditions.

## 3 Results and discussion

### 3.1 Powder bulk characterizations

**Fig. 2:**
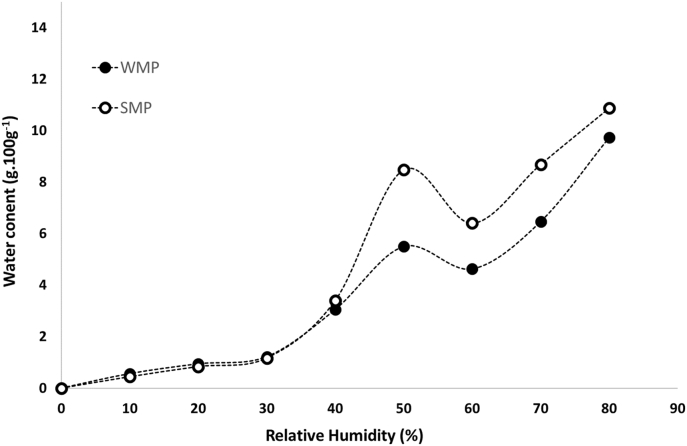
Dynamic Vapor Sorption curves for WMP and SMP.

Glass transition temperatures (Tg) of SMP and WMP, were determined by DSC at various relative humidities. As shown in **Supp 1**, WMP exhibits lower Tg values than SMP, regardless of RH level. This behavior is attributed to the higher fat content of WMP, with probably hydrophobic components reducing the overall Tg of the powder matrix (Gaiani et al., 2026; Saxena et al., 2020). For both powders, Tg progressively decreases as RH increases. As expected, with powder moisture absorption, water acts as a plasticizer which lowers the Tg. Under these conditions, the initial amorphous lactose may undergo crystallization. Water acts as a plasticizer, increasing molecular mobility and facilitating the rearrangement of lactose molecules into a more ordered crystalline form (Afrough et al., 2026). The moisture sorption isotherms of the two powders were determined using DVS (Fig. 2) at 20 °C. Both powders exhibit sigmoidal isotherms characteristic of Type II behavior, a profile commonly observed in food materials (Foster et al., 2005). At low relative humidity (<40% RH), water uptake remains limited for both powders, with a similar behavior, indicating that amorphous lactose retains its initial state with minimal moisture absorption (Phosanam et al., 2020). As relative humidity increases beyond 40%, a sharp rise in water sorption is observed, especially for SMP. This transition zone (40–60% RH) corresponds to the critical moisture uptake phase driven by the hygroscopic nature of amorphous lactose. Water acts as a plasticizer, increasing molecular mobility and promoting conditions favorable to lactose crystallization (Buckton and Darcy, 1995; Sheokand et al., 2014; Jouppila and Roos, 1994). However, this crystallization interval remains broad and not clearly defined, making it difficult to determine precisely which powder begins crystallizing first. The sorption peak is followed by a decrease between 50 and 60% RH, reflecting the partial release of water during the transition to a more ordered crystalline structure (Jouppila and Roos, 1994). The magnitude of this phenomenon differs between the two powders. SMP shows a more pronounced transition, owing to its higher lactose content and low-fat levels, which leave its surface more accessible to moisture (Gaiani et al., 2006; Pugliese et al., 2017). In contrast, WMP exhibits a more gradual sorption profile, as its fat-rich, hydrophobic surface may slow down water adsorption (Maidannyk et al., 2019). Nevertheless, when sorption is expressed on a non-fat basis, the isotherms of SMP and WMP become nearly identical, as previously observed in other studies (Murrieta-Pazos et al., 2011).

### 3.2 Powder surface evolution under controlled humidity

**Fig. 3:**
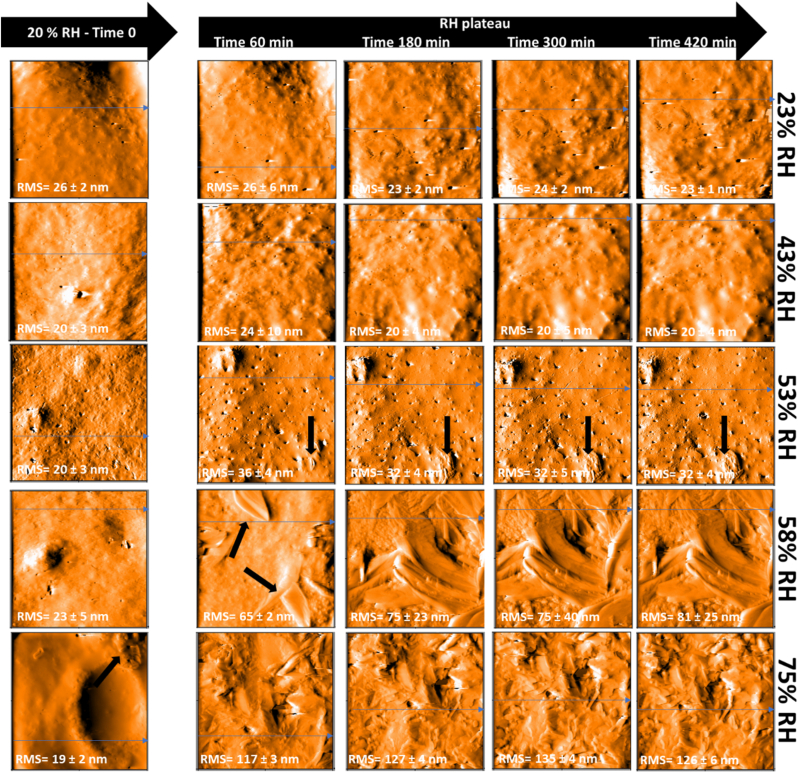
Particle surface evolution for SMP when applying a RH ramp from 20 to 23. 43. 53. 58 and 75% RH respectively. For each RH condition the same particle was analyzed and roughness values were measured at the same location (blue arrow).

**Fig. 4:**
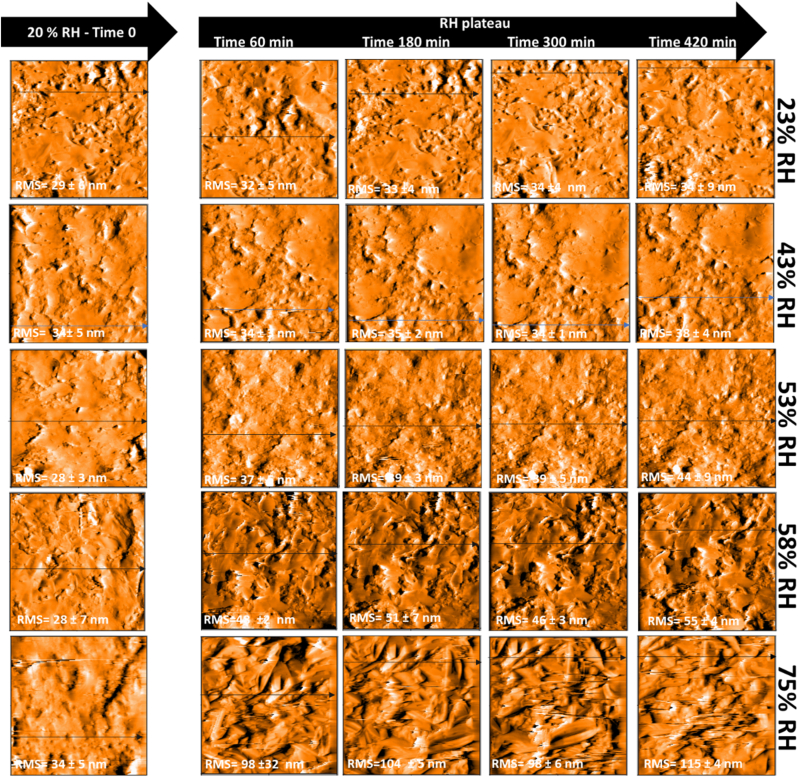
Particle surface evolution for WMP when applying a RH ramp from 20 to 23. 43. 53. 58 and 75% RH respectively. For each RH condition the same particle was analyzed and roughness values were measured at the same location (blue arrow).

**Fig. 5:**
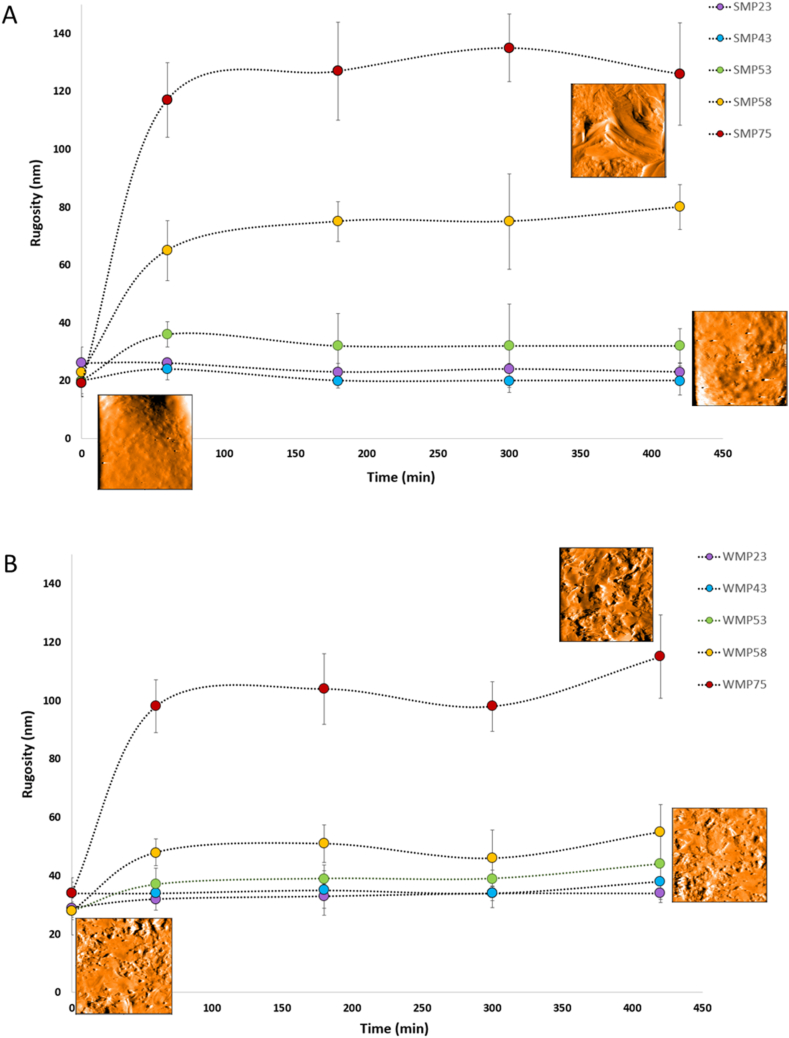
*In-situ* roughness (nm) evolution for SMP (A) and WMP (B) when applying the RH ramp.

**Fig. 6:**
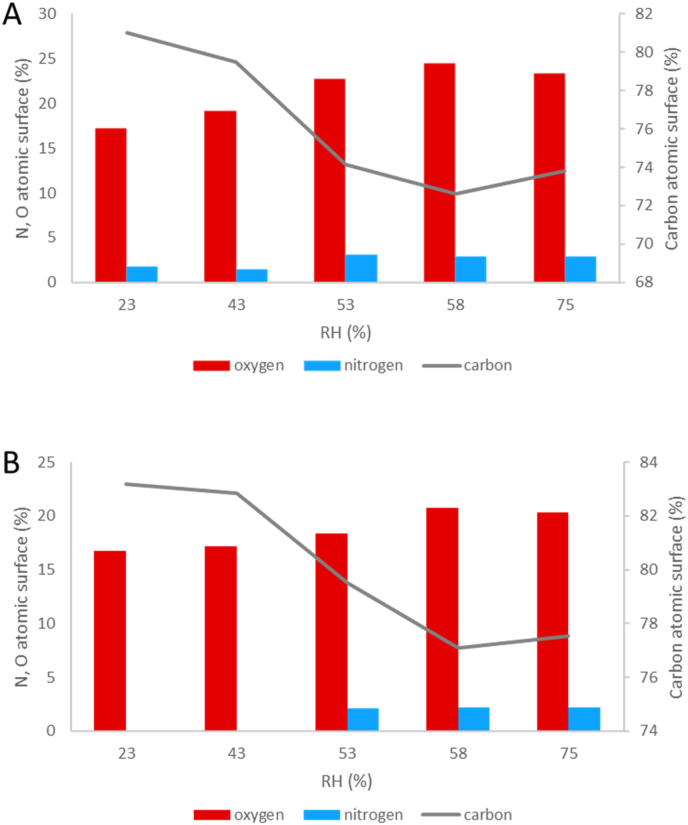
Atomic surface evolution in Carbon, Nitrogen and Oxygen for SMP (A) and WMP (B) when applying the RH ramp.

At low relative humidity (∼20% RH), both SMP and WMP particles present smooth surfaces, but with differences reflecting their distinct compositions. In Fig. 3, SMP exhibits a homogeneous morphology, characteristic of an amorphous lactose-dominated surface (Burgain et al., 2017; Gaiani et al., 2006; Murrieta-Pazos et al., 2011) whereas WMP presents a slightly more heterogeneous surface with micro-domains attributable to fat deposits (Fig. 4) (Phosanam et al., 2021). Roughness values are slightly lower for SMP (19–26 nm) than WMP (28–34 nm) and presented in Fig. 5A and B respectively. These initial topographies, monitored *in-situ* by AFM on single particles are consistent with the surface compositions measured by XPS (Fig. 6). SMP exhibits a lower C/O ratio (∼4.70) reflecting its lactose- and protein-rich surface, while WMP shows a higher C/O ratio (∼4.95), consistent with greater surface lipid coverage (Gaiani et al., 2026).

At 23% and 43% RH, neither powder undergoes significant surface restructuring. SMP roughness remains in the 20–26 nm range without time-dependent evolution, confirming that amorphous lactose maintains its integrity below the critical RH range identified by DVS (40–60% RH, Fig. 2) and consistent with the onset Tg at ∼45% RH (**Supp 1**). WMP shows a modest roughness increase up to ∼38 nm at 43% RH and may be attributable to the slight mobility of surface fat rather than lactose rearrangement. XPS surface composition remains essentially unchanged over this low-RH range for both powders, with stable C/O ratios, confirming the absence of major surface molecular reorganization.

At 53% RH, distinct surface dynamics appear for the two powders. In SMP (Fig. 3, blue arrows), a transition zone is observed with roughness increasing moderately from ∼20 to ∼32 nm, along with the appearance of small nucleation spots on the surface, indicating the onset of moisture-induced plasticization and surface restructuring compatible with early stages of lactose crystallization. At this RH level, XPS data for both powders begin to show a slight reduction in surface carbon and increase in oxygen, consistent with early moisture compositional rearrangements at the outermost surface. In WMP (Fig. 4), a similar onset is detected (∼28–44 nm), but the evolution is less marked and more gradual. While fat-containing powders may exhibit comparable or even earlier onset of crystallization, their growth rate is slower, as lipids can constrain lactose domains and hinder molecular rearrangements (Qí et al., 2025). It was also demonstrated that fat restricts internal lactose rearrangement independently of water transport. Indeed, Qi et al. (Qí et al., 2025) reported that it may be the α-to β-lactose conversion that is delayed rather than moisture uptake itself. The presence of fat in WMP can hinder lactose mobility and translocation within the particle matrix, thereby slowing the progression toward the β-polymorph even when overall water sorption kinetics appear comparable to SMP. Together, these interpretations support previous work showing that the lipid layer in WMP may limit water accessibility and may contribute to delaying the structural transitions associated with lactose crystallization (Phosanam et al., 2021; Qí et al., 2024), while also suggesting that possible lactose mobility restriction may play an additional, complementary role.

At 58% RH, surface rearrangement is pronounced for both powders but proceeds at totally different rates and extents. In SMP, roughness increases sharply in the first 60 min (23 to 65 nm) before stabilizing at 75–81 nm after 420 min (Fig. 5A). Large, distinct surface features emerge, suggesting rapid nucleation and structural reorganization promoted by water sorption, consistent with lactose crystallization (Fig. 3) (Haque and Roos, 2005). In WMP, roughness increases more gradually (29 to 55 nm over 420 min) and to a lower overall extent (Fig. 5B). This may reflect the role of surface lipids on water diffusion and lactose mobility (Qí et al., 2024). These AFM observations are correlated to XPS data in Fig. 6. The C/O ratio decreases significantly in both powders — from 4.70 to 3.17 in SMP and from 4.95 to 3.81 in WMP (**Supp 2**). It should be noted that XPS probes only the outermost ∼10 nm layer and is sensitive to multiple concurrent surface processes (segregation, protein/lipid rearrangement); therefore, the C/O ratio change cannot be attributed solely to lactose crystallization, though it is consistent with this interpretation. Nevertheless, the greater C/O decrease in SMP compared to WMP aligns with differences in crystal morphology as SMP develops larger sheet-like surface features (Fig. 3) than WMP (Fig. 4) (Qí et al., 2024).

At 75% RH, both powders undergo their most dramatic surface transformations. In SMP, roughness increases from 19 to 117 nm within the first 60 min, stabilizing near 126–130 nm. This sharp increase reflects the transition from the glassy to the rubbery state above Tg, which dramatically enhances molecular mobility, promotes inter-particle stickiness and bridge formation, and is subsequently consistent with the development of lactose crystallization (Fitzpatrick et al., 2007; Haque and Roos, 2005; Silalai and Roos, 2010). In WMP, roughness reaches 34 to 115 nm but the surface remains strongly heterogeneous, reflecting presence of fat domains, protein patches, and crystalline structures consistent with distinct surface domains (Kim et al., 2002). Roughness development is lower and slower than in SMP at equivalent conditions.

### 3.3 Effect of RH on physical and functional properties of SMP and WMP

Powder flow properties were characterized using an FT4 powder rheometer and powder sizes by laser diffraction granulometry (Table 2). Particle size analysis revealed differences in median diameter and distribution width between the two powders, with variations in D10, D50, D90 and span suggesting distinct proportions of fine particles and packing potential (Pugliese et al., 2017). These differences were reflected in the CBD, with higher values observed for SMP particles. At low relative humidity (23% RH), the higher CBD of SMP compared with WMP can be attributed to differences in particle size distribution and internal structure, with SMP typically exhibiting smaller particles and lower intraparticle porosity, allowing more efficient packing after conditioning. Upon storage at elevated relative humidity, the CBD of both powders decreases as moisture adsorption promotes capillary bridging, particle swelling and agglomeration, which increase interparticle cohesion and hinder close packing, leading to a looser bulk structure (Teunou and Fitzpatrick, 1999).

**Table 2:** Physical and functional properties of SMP and WMP at different RH.

Powder	Stored RH	FT4 results					Granulometry results			
		BFE (mJ)	SI	FRI	SE (mJ/g)	CBD (g/ml)	D10	D50	D90	span
**SMP**	**23**	553.47 **±** 25.82	0.69 **±** 0.11	0.80 **±** 0.05	2.65 **±** 0.64	0.58 **±** 0.12	63.1	152	299	1.55
	**43**	600.59 **±** 47.39	0.92 **±** 0.03	0.89 **±** 0.06	4.29 **±** 1.83	0.41 **±** 0.13	72.8	171	321	1.45
	**53**	605.51 **±** 0.04	0.90 **±** 0.01	0.90 **±** 0.01	5.37 **±** 0.05	0.33 **±** 0.00	81.3	189	340	1.37
	**58**	670.92 **±** 0.34	0.88 **±** 0.04	1.14 **±** 0.04	6.42 **±** 0.88	0.33 **±** 0.11	88.1	203	378	1.43
	**75**	976.11 **±** 60.75	0.81 **±** 0.06	0.99 **±** 0.02	7.69 **±** 0.04	0.27 **±** 0.01	99.4	208	383	1.36
**WMP**	**23**	518.89 **±** 34.05	1.15 **±** 0.01	0.97 **±** 0.00	3.84 **±** 0.24	0.42 **±** 0.00	70.5	181	390	1.77
	**43**	579.25 **±** 8.52	1.13 **±** 0.01	0.98 **±** 0.02	5.68 **±** 2.51	0.33 **±** 0.12	82.1	228	491	1.79
	**53**	607.90 **±** 23.08	1.13 **±** 0.01	1.04 **±** 0.00	7.73 **±** 0.30	0.23 **±** 0.00	81.5	226	468	1.71
	**58**	753.08 **±** 19.20	1.04 **±** 0.01	1.00 **±** 0.03	9.64 **±** 0.04	0.22 **±** 0.00	86.5	231	478	1.69
	**75**	788.13 **±** 17.03	1.00 **±** 0.04	0.99 **±** 0.05	9.59 **±** 0.23	0.24 **±** 0.00	87.9	236	451	1.54

The evaluation of powders flowability highlights the influence of composition and particle characteristics on powder flow behavior (Fournaise et al., 2020). The BFE, which reflects resistance to flow under confined and stressed conditions, was higher for WMP, indicating a greater opposition to forced movement and suggesting enhanced interparticle friction or consolidation effects, likely related to the presence of surface fat. In contrast, the SE, representative of powder cohesion under low-stress and unconfined flow, differed between the two powders, with higher SE values pointing to stronger interparticle interactions during gentle mobilization. The SI, which assesses the evolution of flow behavior during repeated conditioning cycles, remained close to unity for both powders, indicating limited structural or mechanical changes and thus good flow stability during handling. Finally, differences in the FRI revealed distinct sensitivities of WMP and SMP to flow-rate variations, suggesting that processing conditions such as feeder speed or conveying intensity may differentially impact their flow performance. Increasing RH markedly affected the flow properties of both SMP and WMP, although the magnitude and mechanisms differed between the two systems (Fitzpatrick et al., 2004). For SMP, exposure to elevated RH led to a pronounced increase in SE, indicating enhanced cohesion under low-stress conditions as a result of moisture adsorption on the predominantly hydrophilic particle surfaces and the formation of capillary liquid bridges (Palzer et al., 2010). This increase in cohesion was accompanied by higher BFE, reflecting greater resistance to flow under confined conditions due to moisture-induced consolidation and reduced particle mobility. In contrast, WMP exhibited a more moderate increase in SE and BFE with increasing RH, suggesting that the presence of surface fat partially limited water uptake and delayed the development of strong capillary interactions, although plasticization of the amorphous lactose phase may still contribute at higher humidity levels (Huppertz and Gazi, 2016). The SI of both powders deviated slightly from unity at high RH, indicating progressive changes in structure during repeated testing, consistent with moisture-driven compaction or agglomerate formation. Finally, the FRI increased with RH for both powders, highlighting a growing sensitivity of flow behavior to shear and processing conditions as interparticle adhesion intensified.

### 3.4 From surface restructuring to functional losses

These results converge on a coherent mechanistic picture in which surface composition appears to strongly influence the response of dairy powder to humidity (Gaiani et al., 2026; Burgain et al., 2017). While particle size distribution remains relatively similar between SMP and WMP and does not clearly discriminate their behavior, surface-sensitive techniques reveal marked differences. DVS established the critical plasticization window (40–60% RH) but, when expressed on a non-fat basis, the two isotherms become superimposable (Murrieta-Pazos et al., 2011), confirming that DVS alone cannot discriminate their surface behaviors. DSC quantified the Tg with RH, more advanced in SMP due to its lower fat content (Maidannyk et al., 2019; Silalai and Roos, 2010).

*In-situ* AFM resolved what these bulk techniques could not. At 58% RH, SMP roughness increased approximately 4-fold faster than WMP at equivalent RH conditions. At 75% RH, both powders reached comparable maximum roughness, but WMP surfaces remained heterogeneous, with co-existing fat domains, protein patches and crystalline structures reflecting competing surface dynamics (Kim et al., 2002). XPS provided the surface compositional counterpart: the C/O ratio dropped more steeply in SMP than in WMP, with both reaching a plateau at 75% RH. The higher C/O in WMP confirms the lipid coverage and may explain the possible constrained lactose mobility observed by AFM.

These surface differences were linked to functional properties: between 23% and 75% RH, BFE increased by 76% in SMP versus 52% in WMP, and CBD fell by 53% versus 43%. The steepest changes in SMP occurs precisely in the 53–58% RH window where AFM and XPS both recorded their sharpest transitions (Fitzpatrick et al., 2007; Jouppila and Roos, 1994).

Here, the added value of *in-situ* AFM is to bridge scales and to reveal mechanisms that remain inaccessible to conventional approaches. Positioned as a proof-of-concept, this work demonstrates the potential of real-time surface imaging to refine our understanding of dairy powder behavior under dynamic environmental conditions.

## 4 Conclusion

This pilot study demonstrated the feasibility of *in-situ* AFM under controlled humidity ramps for real-time monitoring of surface evolution in dairy powder particles — applied here for the first time to SMP and WMP. A clear RH-dependent sequence emerged: stable surfaces below 43% RH, onset of roughening above 53% RH, and extensive restructuring at 58–75% RH, compatible with humidity-driven lactose crystallization. DVS, DSC, XPS, and powder rheology corroborated these observations, identifying the critical plasticization window (40–60% RH), confirming Tg depression, revealing concurrent surface compositional rearrangements, and documenting significant flowability losses above 60% RH. SMP restructured earlier and more extensively than WMP, where surface lipids may contribute to delaying plasticization and crystallization kinetics. Future work should extend the approach to broader powder sets, multiple particles, and combined structural analysis to fully validate this characterization framework.

## Funding

This work was supported by Innovation Fund Denmark (AMUSE: Advanced Methodologies to Understand Diary Powder Stability and Promote Exports).

## Declaration of competing interest

The authors declare the following financial interests/personal relationships which may be considered as potential competing interests: Claire Gaiani reports financial support was provided by Innovation Fund Denmark. Serafim Bakalis reports financial support was provided by Innovation Fund Denmark. If there are other authors, they declare that they have no known competing financial interests or personal relationships that could have appeared to influence the work reported in this paper.
