# Supplementary material for: Humidity-driven lactose crystallization on milk powders: a surface-level study
Source: Curr Res Food Sci. 2026 Jun 5;12:101465. doi: 10.1016/j.crfs.2026.101465 (PMC13266239; doi:10.1016/j.crfs.2026.101465)
Supplement: Multimedia component 1 [file mmc1.docx]

**Supplementary material**

**Supp 1.** Glass transition (onset) in °C at different relative humidity’s for the dairy powders.


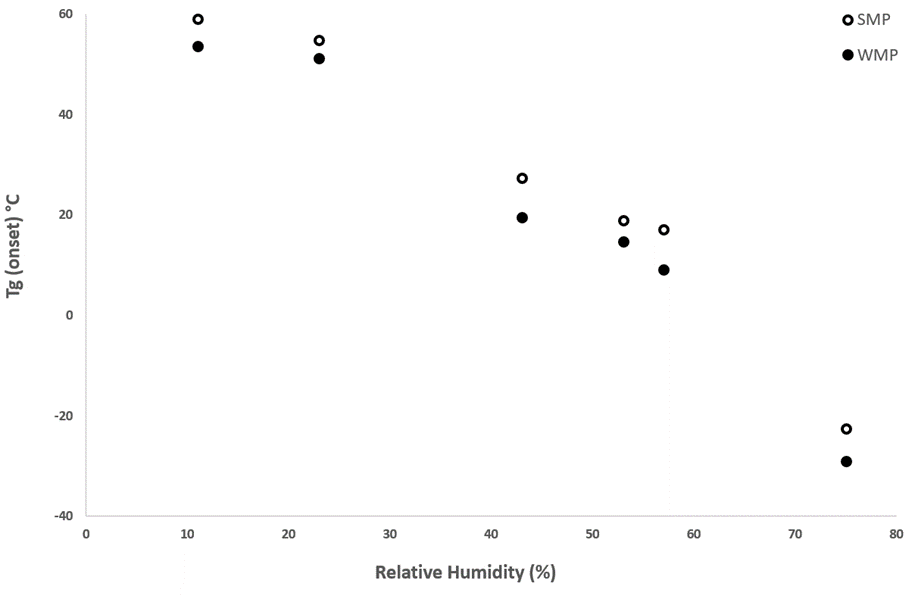


**Supp 2.** Surface atomic percentages for SMP and WMP at each relative humidity.

|  | **Surface atomic composition (%)** | | | | |
| --- | --- | --- | --- | --- | --- |
| **Milk powder** | **RH** | **O** | **N** | **C** | **C/O ratio** |
| SMP | 23 | 17.24**±**0.25 | 1.74**±**0.08 | 81.03**±**0.30 | 4.70**±**0.08 |
|  | 43 | 19.09**±**0.28 | 1.43**±**0.07 | 79.48**±**0.32 | 4.16**±**0.07 |
|  | 53 | 22.73**±**0.35 | 3.12**±**0.10 | 74.15**±**0.38 | 3.26**±**0.06 |
|  | 58 | 24.48**±**0.36 | 2.91**±**0.09 | 72.61**±**0.40 | 2.97**±**0.05 |
|  | 75 | 23.32**±**0.34 | 2.87**±**0.09 | 73.81**±**0.37 | 3.17**±**0.05 |
| WMP | 23 | 16.8**±**0.22 | 0**±**0.00 | 83.2**±**0.25 | 4.95**±**0.07 |
|  | 43 | 17.16**±**0.24 | 0**±**0.00 | 82.84**±**0.27 | 4.83**±**0.07 |
|  | 53 | 18.38**±**0.27 | 2.11**±**0.08 | 79.51**±**0.31 | 4.33**±**0.07 |
|  | 58 | 20.72**±**0.30 | 2.18**±**0.08 | 77.1**±**0.34 | 3.72**±**0.06 |
|  | 75 | 20.36**±**0.29 | 2.19**±**0.09 | 77.54**±**0.33 | 3.81**±**0.08 |
